# Antimicrobial Resistance in the Asia Pacific region: a meeting report

**DOI:** 10.1186/s13756-019-0654-8

**Published:** 2019-12-18

**Authors:** Esabelle Lo Yan Yam, Li Yang Hsu, Eric Peng-Huat Yap, Tsin Wen Yeo, Vernon Lee, Joergen Schlundt, May O. Lwin, Direk Limmathurotsakul, Mark Jit, Peter Dedon, Paul Turner, Annelies Wilder-Smith

**Affiliations:** 1 Centre for Global Health, Lee Kong Chian School of Medicine, Nanyang Technological University, 11 Mandalay Road, Singapore, 308232 Singapore; 2 Saw Swee Hock School of Public Health, National University of Singapore, Singapore, Singapore; 3 Public Health Group, Ministry of Health, Singapore, Singapore; 4 Nanyang Technological University Food Technology Centre and School of Chemical and Biomedical Engineering, Nanyang Technological University, Singapore, Singapore; 5 Wee Kim Wee School of Communication and Information and Lee Kong Chian School of Medicine, Nanyang Technological University, Singapore, Singapore; 6 Mahidol-Oxford Tropical Medicine Research Unit, Faculty of Tropical Medicine, Mahidol University, Bangkok, Thailand; 7 Centre for Tropical Medicine and Global Health, Nuffield Department of Medicine, University of Oxford, Oxford, UK; 8 Department of Infectious Disease Epidemiology, Faculty of Epidemiology and Population Health, London School of Hygiene and Tropical Medicine, London, UK; 9 Modelling and Economics Unit, Public Health England, London, UK; 10 School of Public Health, University of Hong Kong, Hong Kong, SAR China; 11 Antimicrobial Resistance Interdisciplinary Research Group, Singapore-MIT Alliance for Research and Technology, Singapore, Singapore; 12 Department of Biological Engineering, Massachusetts Institute of Technology, Cambridge, MA USA; 13 Cambodia Oxford Medical Research Unit, Angkor Hospital for Children, Siem Reap, Cambodia; 14 Centre for Tropical Medicine and Global Health, Nuffield Department of Medicine, University of Oxford, Oxford, UK; 15 Department of Disease Control, London School of Hygiene and Tropical Medicine, London, UK; 16 Heidelberg Institute of Global Health, University of Heidelberg, Heidelberg, Germany

**Keywords:** Asia Pacific, Singapore, One Health, Global Health, Global Mobility, Travel, Drug Resistance, Antimicrobial Resistance

## Abstract

The Asia Pacific region, home to two-thirds of the world’s population and ten of the least developed countries, is considered a regional hot-spot for the emergence and spread of antimicrobial resistance (AMR). Despite this, there is a dearth of high-quality regional data on the extent of AMR. Recognising the urgency to close this gap, Singapore organised a meeting to discuss the problems in the region and frame a call for action. Representatives from across the region and beyond attended the meeting on the “Antimicrobial Resistance in the Asia Pacific & its impact on Singapore” held in November 2018. This meeting report is a summary of the discussions on the challenges and progress in surveillance, drivers and levers of AMR emergence, and the promising innovations and technologies that could be used to combat the increasing threat of AMR in the region. Enhanced surveillance and research to provide improved evidence-based strategies and policies are needed. The major themes that emerged for an action plan are working towards a tailored solution for the region by harnessing the One Health approach, enhancing inter-country collaborations, and collaboratively leverage upon new emerging technologies. A regionally coordinated effort that is target-driven, sustainable and builds on a framework facilitating communication and governance will strengthen the fight against AMR in the Asia Pacific region.

## Introduction

Antibiotics have revolutionised modern medicine and facilitated advances in transplantation, chemotherapy and surgery, while drastically reducing the mortality and morbidity from infectious diseases. The ever-increasing demand for antibiotics in healthcare is evident in the 65% increase in global consumption from 21.1 billion to 34.8 billion daily doses from 2000 to 2015 [1]. Besides human healthcare, antibiotics are used for the prevention and treatment of diseases in food-producing animals in the agriculture and aquaculture industries, as well as for growth promotion. Medically-important antibiotics accounted for 51% of all domestic sales of antibiotics approved for food-producing animals in the United States (US) in 2017 [2]. The widespread use of antimicrobials has hastened the development of antimicrobial resistance (AMR) through increased selection pressure for genes that confer mechanisms to reduce the effectiveness of antibiotics. This led to the emergence of multi (MDR) and extensively drug-resistant (XDR) pathogens such as *Mycobacterium tuberculosis* (MTB), carbapenem-resistant Enterobacteriaceae (CRE), XDR *Pseudomonas aeruginosa* and *Acinetobacter baumannii* and methicillin-resistant *Staphylococcus aureus* (MRSA). These pathogens are part of the World Health Organisation (WHO)’s priority list published in 2017, widely known as the “dirty dozen” that pose a significant threat to human health [3]. Recognising the potential scale and enormity of the impact of AMR, the Heads of States at the United Nations General Assembly passed a resolution in September 2016 to reaffirm their commitment to tackling the rising threats of AMR [4].

The Asia Pacific region (APAC), home to two-thirds of the world’s population and ten of the least developed countries [5, 6], is highly vulnerable to the threats of AMR. AMR undermines efforts in improving the health systems and health security in APAC and threatens the overall growth potential of the region. In light of the growing threat of AMR in the region, Lee Kong Chian School of Medicine (LKCMedicine) together with Saw Swee Hock School of Public Health; Singapore-MIT Alliance for Research and Technology; National Centre for Infectious Diseases; DSO National Laboratories; and the Ministry of Health, Singapore organised a meeting titled “Antimicrobial Resistance (AMR) in the Asia Pacific & its impact on Singapore”. The meeting was held from 13 to 14 November 2018 at the LKCMedicine in Singapore to coincide with the annual World Antibiotic Awareness Week.

This report summarises the information and insights shared by 26 experts who represented the academic, industry and government sectors from Papua New Guinea, Timor Leste, Indonesia, Thailand, Cambodia, Myanmar, India, Singapore, US and the United Kingdom. This report consists of three sections, namely, the challenges and progress in surveillance, drivers and levers of AMR, and potential innovations and technologies to combat the increasing threat in the region. We will present potential solutions and a roadmap as discussed during the meeting.

### Global antimicrobial resistance

Drug-resistant pathogens have been found in every continent; however, differences between countries in the prevalence of AMR depend on multiple factors including levels of antibiotic consumption, access to clean water, adequate sanitation, vaccination coverage, the availability of quality healthcare, and access to high-quality medical products. Increasing international travel has played a key role in the spread of drug-resistant pathogens including but not limited to MRSA and extended spectrum beta-lactamase (ESBL) producing Enterobacteriaceae [7–9], increased the proportion of drug-resistant enteric pathogens causing travellers’ diarrhoea [10–24], and generally increased the number of travellers infected by drug-resistant pathogens [25, 26]. Globally, third-generation cephalosporin-resistant *Escherichia coli* and *Klebsiella pneumonia* were estimated to cause 3.7 million to 6.4 million bloodstream infections, and 28.9 million to 50.1 million serious infections, and carbapenem-resistant strains caused 0.4 to 0.5 million bloodstream infections, and 2.7 to 3.1 million serious infections in 2014 [27]. In the European Economic Area, five drug-resistant bacterial infections accounted for an estimate of 33,110 attributable deaths and 170 DALYs per 100,000 population in 2015, rivalling the combined burden of influenza, tuberculosis and HIV [28]. These numbers foreshadow the long-term projected consequences of 10 million deaths annually and an additional 24 million people forced into extreme poverty by 2030 if no action is taken against AMR [29–31].

### Antimicrobial resistance in the Asia and Pacific

Asia Pacific is highly vulnerable to the threats of AMR. The challenges that impede the progress in controlling AMR in APAC are wide-ranging and affect both the low-to-middle-income countries (LMICs) and high-income countries. The region is projected to be home to 27 of the world’s 43 megacities by 2030 [32], and these densely populated cities may serve as huge reservoirs for the spread of drug-resistant pathogens. This holds particularly true for LMICs with unplanned urbanisation, creating environments where sanitation is poor, wastewater management is suboptimal, and where air pollution causes respiratory conditions that are often mistakenly treated with antibiotics [1, 32, 33]. Within the region, the WHO South-East Asia (SEA) countries were postulated to have the highest risk of emergence and spread of AMR among all WHO regions [34–36], for which the highly transferable New Delhi metallo-β-lactamase-1 (NDM-1) is a relatively recent reminder [37]. At the same time, the region grapples with a high incidence of infectious diseases. China and India alone account for almost one-third of the global incidence of rifampicin-resistant TB. This number does not include yet another one-third of people infected with TB that are either undetected or not captured by national statistics [38]. Patients in 10 hospitals across India with MDR and XDR bacteria were 1.57 times and 2.65 times more likely to die as compared to similar susceptible infections [39]. In Thailand, 19,122 of 45,209 (43%) deaths in nine hospitals across Thailand were attributed to healthcare-associated infections due to multi-drug resistance [40].

## Antimicrobial Resistance Surveillance Progress and Challenges

The WHO Global AMR Surveillance System (GLASS) and Organisation for Animal Health (OIE) global database on the use of antibiotics in animals were established to enable the systematic collection of data globally. By 2018, 9 of 11 WHO SEA region countries and 6 of 27 Western Pacific Region countries had enrolled into GLASS, but reporting of surveillance data is limited [41]. Complementing the functions of GLASS and OIE in other parts of the world are successful regional AMR surveillance networks such as the European Antimicrobial Resistance Surveillance Network (EARS-Net), Central Asian and Eastern European Surveillance of Antimicrobial Resistance (CAESAR) and Red Latinoamericana de Vigilancia de la Resistencia a los Antimicrobianos (ReLAVRA) [42].

Although several networks collect data on selected pathogens in the region, there are no formal surveillance networks dedicated to AMR in APAC [43]. The lack of standardised and consistent data collection and reporting processes in APAC signifies a massive gap in the ability to reliably determine the burden of AMR and impact of interventions implemented in the region [43, 44]*.* The generation of high-quality data is even more challenging, particularly in settings with limited laboratory infrastructure. In these settings, clinicians frequently prescribe antibiotics without the support of microbiological results and diagnostic specimens are mostly submitted from patients in whom empiric treatments have failed [45], leading to the generation of small datasets with an over-representation of AMR isolates. Empirical therapy is practised even in large “tertiary” hospitals, where the “culture of culturing” among clinicians is frequently lacking [46]. Also, laboratory procedures and quality assurance may be suboptimal due to noncompliance to quality control testing recommendations, resulting in the production of poor quality data [47, 48].

Nonetheless, many efforts have been made to improve the situation. At the regional level, the commitment to address AMR was demonstrated by the inclusion of AMR in the current Association of Southeast Asian Nations (ASEAN) five-year work programme (2016–2020). The signing of the Joint Declaration on Action against AMR by the Heads of States in 2017 and the ASEAN Plus Three Leaders’ Statement on Cooperation against AMR (ASEAN Plus Three are the People’s Republic of China, Japan and Republic of Korea) in 2018 have also sent a clear signal on the recognition of a need for regional collaboration against AMR. Based on the 2017 Joint Declaration, the Philippines, with input from the other ASEAN Member States is preparing a draft Strategic Framework that will guide the ASEAN member states in planning for AMR control. The Strategic Framework is targeted to be endorsed by the ASEAN Health Ministers in the near future and will be followed up by a monitoring & evaluation framework for progress tracking.

At the national level, countries are making progress, albeit at varying pace. Timor Leste, a country that has emerged from a period of political instability and war in recent years, is at the start of the process in establishing a surveillance system to understand the impact of AMR in the country. Although the country’s past has been characterised by weak microbiology laboratory capacity and scarcity of data, the Timorese National Health System has made many efforts in the past few years to improve infrastructure and human resources in microbiology diagnostics and bring together the relevant stakeholders to implement a multi-centric surveillance system by 2019. The progress thus far includes the introduction of antimicrobial susceptibility testing for clinical bacteria isolates and a closer working relationship between the *Laboratorio Nacional de Saude* and National Hospital to incorporate microbiology diagnostics into daily clinical decisions as well as to capture trends of infections and AMR [49]. The collaboration has also generated early evidence indicative of more widespread Gram-negative bacteria AMR in Timor Leste [50].

In contrast, Indonesia, the fourth most populous country in the world, is transitioning from the early phase of its efforts against AMR that started in 2017. Guidelines for surveillance have been developed but not fully implemented across the country, and quality data and analyses are far and few [51]. Nonetheless, the country is in the process of nominating a National Reference Laboratory (NRL) and National Coordinating Centre under the leadership of the Committee of AMR Control. So far, the committee has performed several surveys for ESBL- producing *E. coli* and *K. pneumonia* and other pathogens from blood and urine specimens, and antimicrobial usage (AMU) from hospital prescription in 2016 and 2017. There are opportunities for capacity building through the establishment of external quality assurance and proficiency test in the NRL, and extension of the surveys to the broader network of hospitals including 20 national and province-level referral hospitals, and 100 regional-level referral hospitals. It was also highlighted that the development of guidelines and tools for prescription review would help to raise awareness of AMR and guide decision making to reduce the rate of empirical therapy in Indonesia.

In addition to strengthening AMR surveillance for human health, countries such as Myanmar and Singapore are making progress in incorporating a multisectoral approach in tackling AMR. In Singapore, the National Strategic Action Plan overseen by the AMR Coordinating Office emphasises a One Health approach for education and training, surveillance and risk assessment, and research. In Myanmar, a multisectoral approach combining the expertise of epidemiologists, infectious disease clinicians and social scientists to perform research studies and facilitate translation of evidence to policy for AMR is underway.

### Opportunities for antimicrobial resistance surveillance in the Asia and Pacific

There are several opportunities to improve the capacity for AMR surveillance in the region as a result of new funding streams and technological breakthroughs, building on the foundation laid by past efforts in respective countries and commitment by international and regional bodies. This section briefly covers the potential of technology such as whole-genome sequencing (WGS) before a more extensive discussion on technology in the last section on ***Innovations to Fight Antimicrobial Resistance***.

New funding streams such as the Fleming Fund aim to collate existing AMR data from the laboratories in the region and work prospectively in Indonesia, Laos, Papua New Guinea, Timor-Leste, and Vietnam to build human and laboratory capacity in order to facilitate WHO GLASS data submission [52]. It will augment prior and existing efforts by organisations such as the Wellcome Trust which is supporting many research programmes on AMR in the region, including the development of methods to improve the calculation of drug-resistant infection mortality. Two such methods include:

- The “AutoMated tool for Antimicrobial resistance Surveillance System” (AMASS) application to enable local hospitals to perform data analysis, generate report and share data [53].
- A patient-focused surveillance tool through the “A Clinically-Oriented antimicrobial Resistance surveillance Network” (ACORN) to supplement existing pathogen-focused surveillance systems.

On the technology front, the increasing affordability of WGS presents an opportunity to enhance surveillance efforts by using molecular epidemiology data to map out the evolution, spread and transmission of antibiotic-resistant genes and drug-resistant pathogens. Previous studies that used WGS on *K. pneumoniae* and *Shigellae* spp*.* [54, 55] showed that real-time genetic characterisation could be incorporated into current surveillance programmes to investigate, inform and potentially intervene during outbreaks. These studies have paved the way for the use of WGS on other drug-resistant pathogens such as CRE [56]. Work in this area has been initiated by research groups in Singapore who performed WGS on clinical and surveillance CRE isolates collected from 6 public hospitals and the results have revealed diverse strain types and transmission clusters in Singapore. It also demonstrated the potential to apply WGS to investigate the contribution of plasmids and bacterial strains separately to the transmission of CRE through potential reservoirs, as well as generate molecular data to support conventional epidemiological investigations [57].

Using a One Health approach, samples could be collected from farm to market/restaurant and sequenced to gain insights into the transmission of drug-resistant genes and bacteria from live animals to meat and cooked food, and thus further understand the ecological drivers of AMR. The projects involving WGS will require considerable IT infrastructural investment to process, store and handle a large amount of data; develop cloud-based solutions and visual interface for data sharing; and statistical and bioinformatics expertise for data analysis. It also currently faces challenges in not having an international standardised protocol for sample and data processing, dedicated funding and personnel, and consensus for open communication as well as sharing of samples and data within and beyond countries.

Lastly, building on efforts within countries and acknowledgement of the importance of collaboration with international and regional bodies, stakeholders and policymakers should consider forming a dedicated organisation like a regional Centre for Disease Control and Prevention (CDC). The significant progress achieved by the European CDC and African CDC, including the organisation of the EARS-Net and European Surveillance of Veterinary Antimicrobial Consumption (ESVAC), and launch of the AMR framework to direct efforts and resources to the threats of AMR in Africa, are exemplary of successful regionally coordinated efforts [58, 59]. While signs of progress have been encouraging, the data collection process in APAC could be better coordinated to ensure a comprehensive evaluation of the impact and status of AMR in the region [41, 60]. A regional CDC could provide the focal point to facilitate, support and elevate the AMR agenda above the national level, and help countries to achieve global health targets more effectively.

**Table:** Box 1 Issues, recommendations and target outcomes to improve surveillance on AMR in the APAC

Issues	Recommendations	Target Outcomes
Weak health systems	• Increase country capability and capacity to reliably detect the priority pathogens, and link laboratory results to clinical outcome. • Prescribe microbiological culture (particularly, blood and urine) appropriately. • Report case-based surveillance report, together with evaluating attributable mortality rate for AMR.	Improved capacity and capability to diagnose, treat and prevent AMR at all levels of the health system.
Unclear burden of AMR	• Improve surveillance to better describe the burden of AMR. • Better capture and report records of deaths and other clinical outcomes attributable to AMR. • Develop robust models that are practical and acceptable to policymakers and healthcare providers.	Ability to monitor and evaluate the effects of interventions, and project the impact of AMR using modelling options.
Lack of formal network to address AMR	• Engage policymakers to consider developing an official network for AMR in the region, based on role models developed by European CDC, African CDC and European Medicines Agency, such as EARS-NET and ESVAC.	Consolidation of resources and efforts across countries to deliver impactful programme at the regional level.
Lack of open-access data for global sharing	• Engage with policymakers to make data open-access, such as AMU and AMR surveillance data. • Improve the understanding and utilisation of all surveillance data to decide on resource allocation for interventions and to inform the implementation of action plans.	Robust and reliable data to support further policy engagement, monitoring and evaluating impact of interventions, and research and development.

## Drivers and Levers of Antimicrobial Resistance in the Asia and Pacific

The emerging economies and growing prosperity in APAC will likely exacerbate the trend of AMR. The growth in wealth may lead to an increase in demand for animal protein and the shift to large-scale farming in countries such as China and India which are projected to double antibiotic consumption by 2030 [61]. This trend will be compounded by an increase in purchasing power and access to antibiotics, including new and second-line antibiotics that are expensive [1, 62]. Furthermore, travel to and from APAC is increasing rapidly [63] associated with an increasing influx of drug-resistant pathogens [64]. Non-existent antibiotic stewardship for animal health and wastewater management also contribute significantly to the spread of AMR [35, 43, 65–67].

At the population level, the accrual of risks of drug-resistant infections is not readily perceived by individuals, and at the individual level, the risk of misuse of antibiotics is often overlooked in favour of the potential benefit of recovery from illnesses. For this reason, AMR is often called the “invisible threat”. The AMR risk is further compounded by the lack of awareness of appropriate antibiotic usage. In 2015, a study by WHO revealed widespread antibiotic use across countries, with varying levels of understanding of the appropriate utilisation of antibiotics, and poor understanding of the potential consequences of AMR arising from the misuse of antibiotics in the majority of the respondents [68].

Antibiotics taken without prescription ranged from 9 to 62% in APAC [69]. In Indonesia, almost three-quarters of respondents believe antibiotics could treat colds and flu, and in China, more than half of the respondents reported taking antibiotics in the past six months with 5% of these antibiotics purchased online. These observations are supported by other studies, including one in Australia where consumers visiting pharmacies were misinformed about the role of antibiotics in the treatment of URTIs and other ailments, with over one third believing that antibiotics would cure cold and flu faster [70]. These responses were also found to be related to the propensity for patients to self-diagnose [70].

Due to the increasing public demands for antibiotics, even in outpatient settings, the health care community in Asia increasingly prescribes antibiotics, even if inappropriate, further exacerbated by the absence or weak enforcement of policies for antibiotic stewardship [71, 72]. In Malaysia, private clinics contributed to 87% of the total quantity of antibiotics prescribed in primary care, and the bulk of the prescriptions was given unnecessarily for conditions such as URTI, acute bronchitis, acute gastroenteritis and asthma [73]. The high prescription rate of antibiotics in private clinics was attributed to the tendency for general practitioners to give in to patient’s demand for antibiotics, financial incentives from the sales of the medication, and the lack of understanding of the effectiveness of antibiotics against viral infection such as URTI [73, 74]. The problem is similar in Cambodia, where village physicians, pharmacists and unofficial drug suppliers contribute to unnecessary use of antibiotics [75] due to the preference for the habitual practice of empirical therapy [76], and to compensate for poor infection control [77].

Such practices of self-medication and empirical therapy are driven mainly by poor understanding of the cause of conditions such as UTIs and the association between antibiotic use and AMR. At the same time, health systems in countries such as Singapore and Japan struggle with increasing ageing and immunocompromised populations that are more vulnerable to infections and correspondingly prescribed more antibiotics. The paradox of excessive use of antibiotics and timely access to antibiotics, however, is more prominent in LMICs. The restriction in the use and increase in access to antibiotics, without proper adaption to the local contexts, may have a detrimental effect on the control of infectious diseases and AMR [78].

### Public health campaign and regulatory response

The WHO acknowledged the challenges relating to poor awareness and knowledge of AMR in the WHO Global Action Plan and is leading this front through the World Antibiotic Awareness Week that occurs in November every year [79]. In 2018, the campaign took place in almost all countries in APAC in the form of advocacy activities through mass media and outreach events, education programmes through seminars, rallies and workshops, and creative outlets such as dance competitions and art exhibitions [80].

In addition to health literacy, other socio-behavioural determinants include cultural beliefs and the use of technology. Past studies have found that Asians were more optimistic about disease risks and held stronger fatalistic beliefs about prevention than the European and Americans, potentially explaining the weaker adherence of Asians to prevention recommendations [81]. This suggests a greater need for Asian health campaigns to effectively convey the importance of preventative behaviours such as vaccinations and compliance with proper utilisation of antibiotics. In countries where information and communication technology has permeated the daily lives of its citizens, social media and online influencers are potential nodes for the dissemination of health information. Also, networks and consultations with healthcare professionals could be leveraged to promote appropriate health-seeking behaviours [82]. Such “agents of change” and platforms could be harnessed to deliver public health campaigns and interventions that are culturally relevant and relatable to the target populations. At this stage, many AMR campaigns are in the early phases, and few initiatives have been taken to scientifically measure and assess the effectiveness of the communication strategies. Such evaluation is essential to inform future campaigns and complement them with more in-depth studies of antibiotic utilisation attitudes and behaviours amongst specific populations as well as providers in diverse settings.

The effectiveness of public health campaigns could also be augmented by policy implementation to control the use of antimicrobials where feasible. This may include introducing a requirement for a prescription to buy antibiotics, delinking the prescription and sales of antibiotics to remove financial incentives in the healthcare and food-producing sectors [83–86], and to eventually follow the lead of the European Union which has banned the use of antibiotics for animal growth promotion since 2006 [87]. The US is moving in the same direction, and China will ban the use of antimicrobials for animal growth promotion in aquaculture by 2020 [88]. However, most countries have yet to impose such regulations, and many countries are still at the stage of drafting action plans which will require political will and resources to bring them to reality.

### Economic analyses to strengthen the call for action against antimicrobial resistance

The ability to estimate the financial impact of AMR is essential to justify investment for interventions, especially in LMICs where insufficient resources and competing demands exist. However, current projections of the economic impact of AMR rely greatly on simplified assumptions and data inputs due to limited data on AMR [31, 89, 90]. Many other economic studies have primarily used hospital data on direct costs for treatment, diagnosis and hospitalisation [91], limiting the study of the financial impact of AMR to the hospital setting. Even so, quantifying the excess cost of drug-resistant infections in the hospital setting has proven to be difficult as the use of different methodologies in different studies has generated a broad range of excess costs ranging from hundreds to hundreds of thousands of US dollars per patient. Any projection should also be used with caution considering methodological limitations such as external validity due to the heterogeneity in caseloads, care patterns, study populations and pathogen distribution across the populations [91–93].

More importantly, hospital data does not account for the pervasive impact AMR has on society and the future. A broader assessment including the loss of productivity due to morbidity and mortality arising from AMR, loss of revenue in travel and trade due to fear of infections [94], reduction of productivity and wellbeing due to a reluctance for high-risk medical procedures, and the financial consequence of general adverse psychological effects such as panic on the health and wellbeing of the population should be considered. The increasing prevalence of AMR and possible reversion to the pre-antibiotic period when infectious diseases are more deadly and invasive procedures such as elective surgery are more dangerous to perform should also be factored into the calculation of the global cost of AMR [95].

**Table:** Box 2 Vaccination in the War Against AMR

By reducing the need for antibiotics, vaccines may reduce the prevalence and hinder the development of resistant strains. Introduction of a conjugate pneumococcal vaccine for infants in the US in 2000 saw a 57% decline in invasive disease caused by penicillin-resistant strains and a 59% decline in strains resistant to multiple antibiotics by 2004 across a broad age range - 81% among children under 2 years of age and 49% among persons aged 65 years and older [96]. Similarly, in Korea, the serotypes covered by the 7-valent pneumococcal conjugate vaccine (PCV7) was reported to show a 9.3% decline in resistance to cefotaxime and 11% decline in multi-drug resistance after the introduction of the vaccine in the period 2004 to 2008, as compared to the period before between 1996 to 2003. In contrast, the serotypes not covered by PCV7 showed an increase in resistance to cefotaxime by 9.5% and multi-drug resistance by 15.6% [97]. These results highlight the potential of using existing vaccines and developing new vaccines to tackle AMR. Vaccines against viruses such as influenza or dengue also have a role to play as it reduces the incidence of viral infections that are often mistakenly treated with antibiotics. So far, there have been few studies that quantify the effect of vaccines on AMR dynamics, suggesting a gap which could be filled by mathematical models to understand the impact of vaccination on the transmission of drug-resistant pathogens [98].	

## Innovations to Fight Antimicrobial Resistance

### Innovations in diagnosis and surveillance of antimicrobial resistance

The earlier section ***Antimicrobial Resistance Surveillance*** touched on the potential of technology such as WGS to understand the transmission dynamics of drug-resistant genes and pathogens and highlighted the potential for new therapeutic, diagnostic and surveillance innovations to bolster the fight against AMR.

One of the most promising areas in which technological innovations can be used to tackle AMR is to minimise empirical therapy, which has contributed to the unnecessary prescription of antibiotics for viral infections such as URTI. Among the factors promoting the practice of empirical therapy is the lack of affordable and simple point-of-care (POC) diagnostics that can distinguish bacterial from other infections and readily determine the antimicrobial susceptibility for the former. A variety of approaches to developing novel POC diagnostic tools have emerged to increase the throughput, speed, and cost-effectiveness to diagnose infectious diseases and determine the antibiotic susceptibility profile. Recent new developments include the combination of microfluidics to integrate DNA and RNA extraction, and PCR amplification with technologies such as micro-optics to create optofluidic platforms to enable single-cell analysis and pathogen identification [99, 100]. Another success is the development of ultra-fast microfluidics-based chips that work with low number of cells for liquid-phase DNA/RNA purification and subsequent PCR amplification in a few minutes [101, 102]. These have the potential for customisation of infectious disease panels that correspond to different disease burdens in different parts of the world.

The application of engineering design principles to create affordable, fast, and accurate medical diagnostics is also gaining prominence [103–105]. This is illustrated in the application of the principles of immunochromatographic rapid diagnostic tests in over-the-counter pregnancy tests to create affordable POC diagnostics for infectious disease [103]. Given the problems of thermal denaturation and nonspecific binding events that occur with antibody-based tests, robust affinity reagents based on thermostable protein scaffolds can be complemented with systematic approaches to enhance assay sensitivity and ease-of-use [103–105].

Another potential development that could complement pathogen-specific tests is the use of systems-level “omic” analyses that profile host-derived response markers that are predictive of the infections. Initial successes in using integrated omic analyses to identify human biomarkers have been shown in studies that accurately differentiate sepsis from systemic inflammation [106], differentiate mild and severe forms of dengue infection [107], and predict host-pathogen interactions [108].

### Innovations in pre-clinical and regulatory science to reduce antimicrobial development costs

In addition to diagnostics and surveillance, innovative approaches could also help to overcome the barriers in developing new drugs. The high cost of research and development and uncertainties in the return of investments have caused most large pharmaceutical companies to abandon development of new antimicrobials [109, 110], shifting the challenge to academic researchers and smaller biotechnology and pharmaceutical companies. One of the ways to reduce the cost of development is to reduce the development timeline by borrowing lessons learnt from biological therapeutics. This includes a two-pronged approach involving the multiplexing of pre-clinical and potentially clinical analytics with “omics” technologies, and dovetailing drug discovery, development, and manufacturing processes [111]. The combination of these efforts has the potential to reduce the timeline, and thus the cost, for pre-clinical development of a new antimicrobial agent from years to months. Similar innovations in manufacturing and other processes [112], as well as approaches to drug discovery such as repurposing [113], may further reduce the costs of the development phase and promote existing efforts to incentivise investments in new antimicrobials [114].

### Innovations in studying and manipulating the human microbiome for AMR diagnosis and treatment

Understanding of the human microbiome as being important in the establishment of reservoirs of drug-resistant pathogens is emerging [115, 116]. The microbiome is also a potential target for novel approaches to controlling AMR [23, 117–122]. This is most apparent in the technological innovations that are advancing our understanding of the role of the human microbiome in both acquiring and combating AMR [123, 124]. These innovations are driven by the goals of not only identifying and quantifying each of the thousands of types of bacteria, bacteriophages, and other organisms in each human organ, but also of culturing and storing the isolated organisms, some of which are fastidious anaerobes.

The latter problem is being addressed by a team of scientists that founded the Global Microbiome Conservancy, which has the goal of culturing and sequencing more than 10,000 bacterial strains from the human gut in populations from more than 30 countries (http://microbiomeconservancy.org). Building on an initial collection of 7600 gut bacterial isolates and their genomes from healthy and urban North Americans, the Global Microbiome Library expanded the collection with more than 4000 additional strains from under-represented populations in Arctic regions, and East and Central Africa. Defining the genomic sequences and the presence of AMR genes in this growing strain collection has relied on recent innovations in WGS technology, while the identification of sequences of individual microbes among the thousands in a single faecal DNA sample requires even more sophisticated sequencing innovations, such as epicPCR for profiling genetic traits such as AMR [125]. By necessity, these sequencing innovations are being matched with computational innovation for assembling and mining genomes of gut, skin, and other microbial communities, including identifying and quantifying AMR genes [126].

One of the success stories arising from innovations in microbiome science has been the use of faecal microbiome transplants (FMTs) to treat chronic, drug-resistance infections by *Clostridium difficile*. Overgrowth of this anaerobic, sporulating, Gram-positive bacillus following the use of broad-spectrum antibiotics for treating other infections has become a major health threat in the past two decades with the emergence of a particularly virulent strain of *C. difficile* [127]. Previously treatable with a variety of antibiotics, 25% of *C. difficile* infections relapse one or more times and require multiple prolonged courses of metronidazole or vancomycin. However, FMTs have proven curative in up to 90% of relapsed cases in several studies [127], presumably by repopulating the gut with a “healthy” set of microbes that outcompete the *C. difficile*. This same approach can potentially be explored for AMR, with FMT as a major therapeutic tool in de-colonising intestinal carriage of drug-resistant genes and bacteria [128].

**Table:** Box 3 Innovations to address AMR

**Surveillance:** • Harmonise sample and data collection procedures and enable access to samples and data for testing and mining. • Adopt molecular epidemiology using whole-genome sequencing to complement phenotypic studies for transmission dynamics. **Diagnostics:** • Develop rapid diagnostic or point-of-care diagnostics for quick identification of pathogens and antimicrobial susceptibility. • Develop non-pathogen-based approaches that exploit human response signatures to complement existing pathogen-targeted technologies. **Therapeutics:** • Collaborate with regulatory agencies to shorten the timeline for drug development at all stages, from target discovery to clinical trials, thus reducing the cost of developing new AMR therapeutics. • Develop new vaccine and antibiotic pipelines, and explore innovative approaches such as immunotherapy, phage therapy, drug repurposing, and combination therapy.	

## Conclusion

Three recurring themes emerge from the discussions above. The first is that there is no single solution or path to AMR control in the region, given the socioeconomic and cultural diversity within APAC, rapid changes in the region, and the multifaceted nature of the challenges of AMR. The second is the need for multisectoral and One Health approaches to synergise the efforts and harness the expertise and experiences in surveillance, sociobehavioural, economics and innovations. The third is the value of inter-country collaborations in the region and the necessity to elevate the issue of AMR control beyond just national agendas. After all, progress achieved in one country could be off-set by failures in another country. It is an opportune time to put together a regionally coordinated effort that is target-driven, sustainable, and builds on a framework that facilitates communication and consistent governance to strengthen the fight against AMR.

## Contribution to existing literature

This report neither provides an extensive review of AMR in the region which has been captured exceptionally well in existing literature [35, 43] nor covers AMR in the context of topics such as financing and global health security. Nonetheless, it adds to existing literature the voices of people across the region who work in infectious disease control, advocacy and research. These people shared the recognition of the growing threat of AMR and the keen interest in promoting the agenda of AMR by bringing to wider attention the issues and drivers of AMR and garnering support and coordination within and beyond institutions and countries.

## Data Availability

No supporting data to be provided.

## Abbreviations

AMR: Antimicrobial resistance
AMU: Antimicrobial usage
APAC: Asia Pacific region
ASEAN: Association of Southeast Asian Nations
CAESAR: Central Asian and Eastern European Surveillance of Antimicrobial Resistance
CDC: Centre for Disease Control and Prevention
CRE: Carbapenem-resistant Enterobacteriaceae
EARS-Net: European Antimicrobial Resistance Surveillance Network
ESBL: Extended spectrum beta-lactamase
ESVAC: European Surveillance of Veterinary Antimicrobial Consumption
FMTs: Faecal microbiome transplants
GLASS: Global AMR Surveillance System
LKCMedicine: Lee Kong Chian School of Medicine
LMICs: Low-and-middle-income countries
MDR- and XDR MTB: Multi-and extensively drug-resistant *Mycobacterium tuberculosis*
MRSA: Methicillin-resistant *Staphylococcus aureus*
NRL: National Reference Laboratory
OIE: World Organisation for Animal Health
PCV7: 7-valent pneumococcal conjugate vaccine
POC: Point-of-care
ReLAVRA: Red Latinoamericana de Vigilancia de la Resistencia a los Antimicrobianos
SEA: South East Asia
URTI: Upper respiratory tract infection
US: United States
WGS: Whole Genome Sequencing
WHO: World Health Organisation
